# Canakinumab for the treatment of autoinflammatory very early onset- inflammatory bowel disease

**DOI:** 10.3389/fimmu.2022.972114

**Published:** 2022-09-20

**Authors:** Eliana Shaul, Máire A. Conrad, Noor Dawany, Trusha Patel, Megan C. Canavan, Alyssa Baccarella, Sarah Weinbrom, Daniel Aleynick, Kathleen E. Sullivan, Judith R. Kelsen

**Affiliations:** ^1^ Division of Gastroenterology, Hepatology, and Nutrition, Department of Pediatrics, The Children’s Hospital of Philadelphia, Philadelphia, PA, United States; ^2^ Department of Pediatrics, Perelman School of Medicine at the University of Pennsylvania, Philadelphia, PA, United States; ^3^ Department of Biomedical and Health Informatics, The Children’s Hospital of Philadelphia, Philadelphia, PA, United States; ^4^ Division of Allergy and Immunology, The Children’s Hospital of Philadelphia, Philadelphia, PA, United States

**Keywords:** canakinumab, very early onset-inflammatory bowel disease, interleukin-1, pediatric, autoinflammatory

## Abstract

**Introduction:**

Therapeutic options are critically needed for children with refractory very early onset inflammatory bowel disease (VEO-IBD). Our aim was to evaluate clinical response to canakinumab, an anti-IL-1β monoclonal antibody, in patients with VEO-IBD whose phenotype resembles those with monogenic autoinflammatory disease.

**Methods:**

This is a single center retrospective study of patients with VEO-IBD with autoinflammatory phenotype (AIP) in the absence of identified monogenic disease treated with canakinumab for >6 months. AIP was defined as confirmed IBD with associated signs of systemic inflammation in the absence of infection, including leukocytosis, markedly elevated inflammatory markers, and extraintestinal manifestations (recurrent fevers, oral ulcers, arthritis). Primary outcomes included clinical response in disease activity indices after 6 months of therapy. Secondary outcomes included rate of AIP signs and symptoms, growth, surgery, steroid use, hospitalizations, and adverse events.

**Results:**

Nineteen patients were included: 47% with infantile onset, 58% classified as IBD-U, and 42% classified as CD. At baseline, 37% were biologic naïve, and canakinumab was used as dual therapy in 74% of patients. Clinical response was achieved in 89% with statistically significant improvement in PCDAI and PUCAI. Clinical remission was achieved in 32% of patients. There was significant improvement in the clinical manifestations of AIP and the biochemical markers of disease. Number of hospitalizations (p<0.01) and length of stay (p<0.05) decreased. Growth improved with median weight-for-length Z-score increasing from -1.01 to 1.1 in children less than 2 years old. There were minimal adverse events identified during the study period.

**Conclusion:**

Canakinumab may be an effective and safe treatment for a subset of children with VEO-IBD with AIP, as well as older patients with IBD. This study highlights the importance of a precision medicine approach in children with VEO-IBD.

## Introduction

Inflammatory bowel disease (IBD) is a chronic inflammatory disease of the intestinal tract. The etiology involves the complex interaction between the host immune response to environmental exposures in genetically susceptible individuals. Very early onset (VEO)-IBD, a term which describes a growing population of children who present with the disease at less than six years of age, represents a unique form of the disease (1). These patients can be phenotypically and genetically distinct from older children (pediatric onset IBD) and adults who develop IBD. While older onset IBD is most frequently a polygenic disease, monogenic defects have been identified in a subset of patients with VEO-IBD. These discoveries have shed light on the diverse causal pathways and subsequent heterogenous phenotypes in children with VEO-IBD, transforming therapeutic options and overall clinical care. However, while more than 80 genes have associated with VEO-IBD, these findings remain rare and most patients do not have an identified genetic defect (1–3)..

The disease course in children with VEO-IBD can be mild or similar to pediatric and adult onset IBD, however a subset of children can present with a distinct phenotype and have a more aggressive and refractory disease (4). For example, patients with VEO-IBD have higher rates of growth failure and poorer response to anti-tumor necrosis factor (TNF)-α therapy as compared to pediatric onset IBD (5). Furthermore, in addition to varying degrees of severity, there is tremendous phenotypic heterogenicity within VEO-IBD, ranging from isolated intestinal symptoms to intestinal disease with evidence of immune dysregulation or immunodeficiency to systemic manifestations of inflammatory disease. Therefore, in the absence of a monogenic defect and clear etiology of disease, novel approaches are critically needed to identify therapeutic targets for the individual patient to achieve remission. One potential strategy is to apply therapies used successfully in children with identified monogenic disease to those with similar phenotypic characteristics but with an unrevealing genetic evaluation.

An example of this approach is the use of interleukin (IL)-1β blockade in a subset of children with VEO-IBD. The IL-1 family of cytokines, including IL-1α and IL-1β, and the receptor antagonist, IL-1Rα, is associated with pro-inflammatory responses (6). IL-1β has several roles, including chemotaxis through recruitment of neutrophils and stimulation of effector innate cells, such as dendritic cells and macrophages (7). Moreover, IL-1β promotes the activation of proinflammatory T-cells, while IL-1 signaling is necessary for CD4 T cell effector function (8, 9). As a result of these properties, activation of IL-1β plays a central role in several autoimmune and autoinflammatory diseases (AID), a heterogenous group of systemic inflammatory disorders that involve dysregulation of the innate immune response. Monogenic defects have been identified in some forms of AIDs, including defects resulting from mutations in proteins that control caspase-1, an enzyme that can convert pro-IL-1β to its active form (10). In these processes, IL-1β, along with IL-18 and other pro-inflammatory cytokines, is over produced from the activation of the inflammasome, a large innate immune complex and leads to increased inflammation (11, 12). A targeted approach with IL-1 receptor antagonist, anakinra, or human anti- IL-1β monoclonal antibody, canakinumab, have been proven to be efficacious in some AIDs. Canakinumab is approved for treatment of a variety of AIDs including Still’s disease, familial Mediterranean fever (FMF), mevalonate kinase deficiency (MVK), tumor necrosis factor receptor–associated periodic syndrome and cryopyrin-associated periodic syndromes (CAPS), such as familial cold autoinflammatory syndrome and Muckle-Wells syndrome (13, 14). In addition to its use in hereditary autoinflammatory diseases, IL-1 blockade has been promising as a treatment for other inflammatory conditions such as heart failure, acute gout flares and cancer (15, 16).

The application of IL-1β blockade to VEO-IBD stems from the overlapping disease process and phenotype of AID in a subset of patients with VEO-IBD. Indeed, some of the above defects can present with intestinal disease (17). Clinical features include systemic involvement with fevers, oral aphthous ulcers, arthritis, rash or markedly elevated inflammatory markers, along with gastrointestinal symptoms. IL-1β blockade has been used successfully in patients with VEO-IBD with identified variants in known AID genes, such as *MEFV*, *MVK* and *NLRC4* (10, 18–21). Additionally, IL-1β blockade has also been used for other indications in patients with VEO-IBD, including as bridge to hematopoietic stem cell transplantation in patients with IL10 signaling defects, chronic granulomatous disease (CGD) resulting in phagocyte abnormalities and T regulatory defects (17), among others. Importantly, canakinumab has shown an excellent safety profile with an overall good tolerance and few adverse effects (22). Given the efficacy of canakinumab in AID (including patients with VEO-IBD with monogenic AID), we applied this therapy in a subset of patients with VEO-IBD who have an autoinflammatory phenotype (AIP) in the absence of an identified causative defect. Here we describe our experience with this approach.

The primary aim of this study was to evaluate for clinical response and clinical remission in children with autoinflammatory VEO-IBD treated with canakinumab. The secondary aims were to study the tolerability and safety of canakinumab in children with VEO-IBD and to assess improvements in the characteristics of AIP and other clinical markers of disease.

## Materials and methods

### Patient population and study design

This is a single-center, retrospective study of patients with VEO-IBD with an AIP treated with canakinumab. Inclusion criteria were diagnosis of IBD at less than six years of age, AIP, primary IBD care provided at The Children’s Hospital of Philadelphia (CHOP) VEO-IBD center, and treatment with canakinumab, as monotherapy or combination therapy, for a minimum of six months from January 2018 through March 2022 with complete data available for review. AIP was defined based on the consensus proposal for taxonomy and definition of AIDs (23) as dysregulation of the innate immune system, characterized by recurrent or continuous inflammation with elevated acute phase reactants and the lack of a primary pathogenic role for the adaptive immune system. This includes leukocytosis (white blood cell count greater than the upper limit of normal for age), markedly elevated inflammatory markers at least two times the upper limit of normal, and extraintestinal manifestations (EIMs) including recurrent fevers defined as at least three episodes of fever over one month in the absence of infection, oral ulcers and joint pain. Exclusion criteria included patients with identified causative monogenic defects for VEO-IBD and AID, patients whose primary GI care was not at CHOP, therapy course less than six months, canakinumab prescribed for an indication other than AIP or incomplete available data at six months. Data were collected from the electronic medical record and included demographics, age of disease onset, location of disease, disease phenotype, EIMs, growth parameters and nutritional status, laboratory data, cytokine analyses, prior medications, surgical history, hospitalizations, packed red blood cell (pRBC) transfusion requirement, canakinumab dosing and adverse events. Genetic testing and clinical immunophenotyping (evaluation of B cells, T cells, natural killer cells, dihydrorhodamine (DHR) flow cytometric analysis to evaluate granulocyte oxidative bursts, quantitative immunoglobulins, and vaccine titers) were performed on all patients prior to the initiation of canakinumab. Disease activity was measured using the Pediatric Crohn Disease Activity Index (PCDAI) or Pediatric Ulcerative Colitis Activity Index (PUCAI) scores, which were calculated at baseline, within one month of initiation, after 3 months of therapy when available and at six months. Malnutrition was defined using the WHO criteria based on weight-for-length and BMI Z-score for age. Severe malnutrition was defined as Z-score of -3.0 or less, moderate malnutrition as Z-score of -2.99 to -2.00 and mild malnutrition as a Z score of -1.99 to -1.00.

### Outcome measures

Primary study outcomes were clinical response and clinical remission after six months of therapy with canakinumab. Clinical response was defined as a decrease in PUCAI score by at least 20 points in those with ulcerative colitis (UC) or inflammatory bowel disease unclassified (IBD-U) or a decrease in PCDAI score by 12.5 points or greater in subjects with Crohn disease (CD). Outcome measures in patients with diverting ileostomies at baseline included assessment of ostomy and rectal output. Clinical remission was defined as inactive disease with a PUCAI <10 in patients with IBD-U or a PCDAI ≤10 in those with CD and no current corticosteroid therapy. Secondary study outcomes included clinical response and remission at 3 months, measured improvement of signs and symptoms of AIP including EIMs and change in markers of inflammation including white blood cell (WBC) count, C-reactive protein (CRP), and erythrocyte sedimentation rate (ESR). Secondary outcomes also included biochemical markers of disease including hematocrit (hct), and serum albumin, height, weight and weight-for-length or BMI Z score for age, adverse events, hospitalizations, corticosteroid use, surgical outcomes, and transfusion requirement. Laboratory data was obtained at baseline, three months when available and at six months. Normal laboratory values were determined by reference ranges based on age and sex at the laboratories at CHOP or the testing agency.

### Statistical analysis

Lab values below the limit of detection (LOD) were imputed by dividing the LOD over the square root of two. The Wilcoxon signed-rank test was performed to compare continuous variables at baseline and six months. For categorical data, Fisher’s exact test was used. Statistical significance was set at a p-value less than 0.05. Continuous and categorical variables are presented as median (range) and number (percentage), respectively, throughout the manuscript.

## Results

**Figure 1:**
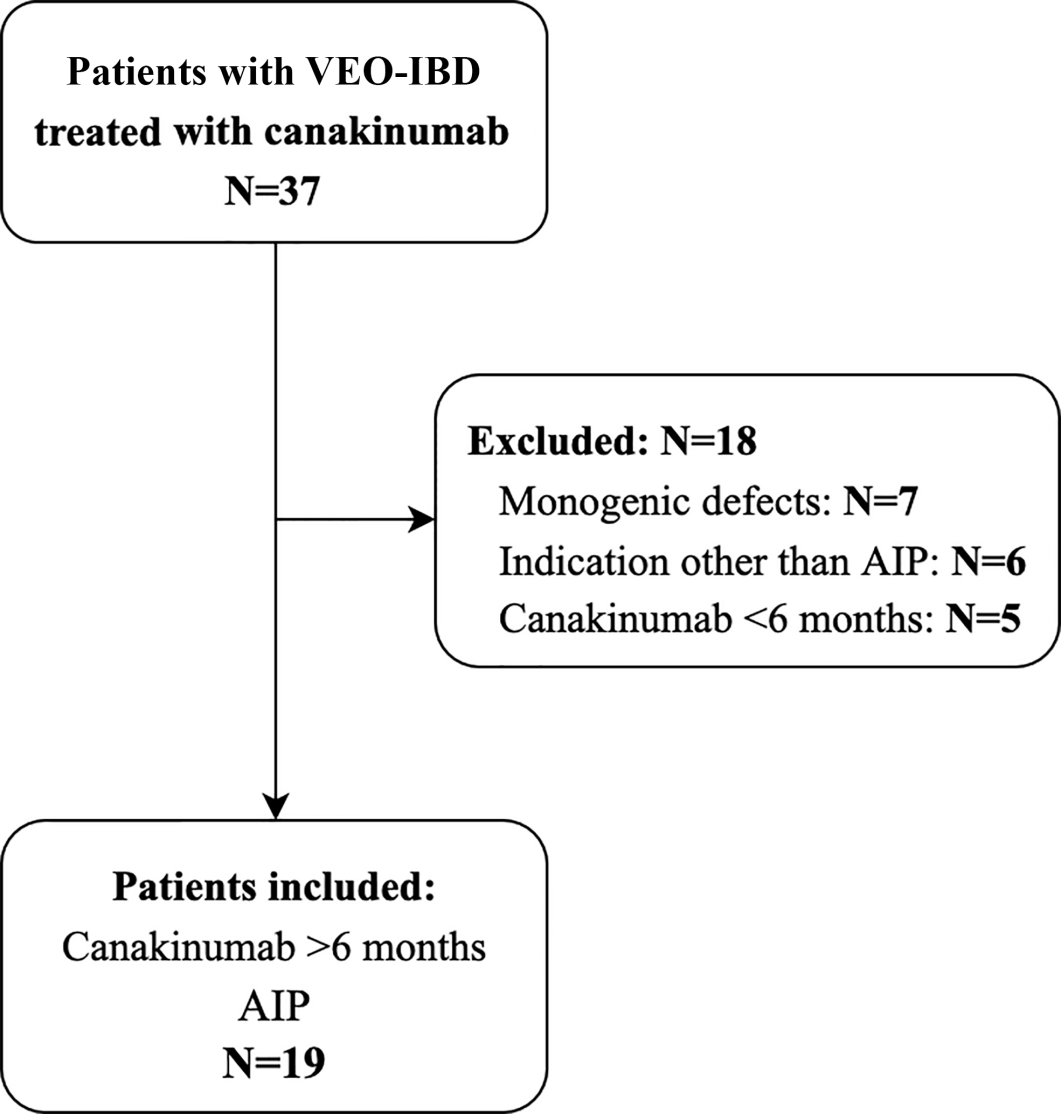
Flowchart of patient inclusion and exclusion.

Thirty-seven children with VEO-IBD treated with canakinumab at CHOP were identified during the study window, of whom 19 met inclusion criteria. Excluded patients were those with identified monogenic defects (N=7), those whose indication for treatment was other than AIP (N=6) and those who did not meet the study duration criteria of 6 months (N=5) (**Figure 1**).

The median age of VEO-IBD diagnosis of this study cohort was 1.83 years (range 0.02-6 years), including nine (47%) with infantile-onset IBD, diagnosed at less than 1 year of age. Eleven (58%) were classified as IBD-U, with the remaining eight (42%) classified as CD. There were no patients with a diagnosis of UC included. Canakinumab was initiated at a median age of 2.2 years (range 0.3-13.1 years). The demographics and baseline characteristics of the 19 patients included in this study are outlined in **Table 1**.

**Table 1:** Clinical Characteristics at Initiation of Canakinumab Therapy (n=19).

	
Male, n (%)	9 (47)
**Age at diagnosis, n (%)**	
<1 year	9 (47)
1 to <6 years	10 (53)
**Age at canakinumab initiation, n (%)**	
<1 year	7 (37)
1 to <6 years	7 (37)
6 to <10 years	4 (21)
≥10	1 (5)
**IBD subtype, n (%)**	
IBD-U	11 (58)
CD	8 (42)
**Disease Phenotype, n (%)**	
Inflammatory only	18 (95)
Stricturing	1 (5)
**Disease Location, n (%)**	
Colonic	15 (83)
Pan-enteric	2 (11)
Ileocolonic	1 (6)
Perianal involvement	3 (17)

Targeted genetic sequencing and whole exome sequencing were performed on all patients and parents without any identified definitive causative mutations in genes associated with VEO-IBD, other inflammatory diseases or any known human diseases. Similarly, immunophenotyping was performed on all patients prior to initiation of therapy and did not reveal specific immunodeficiencies in any patient. DHR testing was not diagnostic of CGD in any patients. Defects in adaptive immunity were not identified on lymphocyte subset or in qualitative immunoglobulins analyses. One patient had mildly low IgG in the setting of hypoalbuminemia which was not clinically relevant (**Supplementary Table 1**). Cytokine analysis was performed on 12/19 (63%) patients. Due to the retrospective design of this study, the varying cytokines were studied throughout the study window. IL-1β was mildly elevated in 3 patients, but along with IL-18 was not universally elevated (**Supplementary Table 2**).

### AIP characteristics

Characteristics of AIP at baseline included EIMs, leukocytosis and elevated markers of inflammation. Regarding EIMs, all patients had recurrent fevers (as defined in Methods) during the six-month period prior to initiation of canakinumab. Oral ulcers were present in 47% and arthritis in 32% of patients, respectively. Elevated inflammatory markers and/or leukocytosis were present in all patients, with a median WBC of 19.1 K/uL (7.2-29.5) prior to corticosteroid use, ESR of 54 mm/hr (4-130, normal <20) and CRP of 3.8 mg/dL (<0.5-25.6, normal <1).

### Baseline disease activity

The baseline disease activity was moderate to severe in 15 (79%) patients and mild in four (21%) patients (median PUCAI: 45 (25–65), PCDAI: 37.5 (27.5-55)). Prior surgical intervention included diverting ileostomy in seven (37%) patients, followed by sub-total colectomy in two patients. Five of the seven patients with a surgical history had an ileostomy in place at the time of canakinumab initiation. Five (26%) patients in the cohort were on steroids at baseline, and seven other children (37%) had a prior history of steroid dependency. Secondary measures of disease severity included number and length of hospitalizations, with 16/19 (84%) of patients hospitalized within six months prior to canakinumab initiation, (median 11 days (0–102),). Moderate malnutrition was present in three (16%) patients and four (21%) patients met criteria for mild malnutrition. Five (26%) patients were total parenteral nutrition (TPN) dependent, and thirteen (68%) patients required supplemental enteral feeds *via* either gastrostomy (G) tube or nasogastric (NG) tube at baseline. In addition to the inflammatory markers described above, other biochemical markers indicative of severity of disease of this cohort included 11 (57.9%) patients with hypoalbuminemia (1.6-4.6 g/gL) and 17 (90%) with anemia (hct 22.1-42%), 11 (58%) of whom were transfusion dependent. Prior failed therapies included biologics (anti-TNFα therapy, vedolizumab and ustekinumab) in 12 (63%) patients, of whom two (17%) had failed one biologic therapy and ten (83%) had failed two or more biologics or immunomodulators. Baseline disease severity is summarized in **Table 2**.

**Table 2:** Baseline Disease activity and severity (n=19).

	
PUCAI, n =11	
Mild (PUCAI 10-34)	2 (18)
Moderate (PUCAI 35-64)	8 (73)
Severe (PUCAI >65)	1 (10)
**PCDAI, n=8**	
Mild (PCDAI 10-30)	2 (25)
Moderate to Severe (PCDAI >30)	6 (75)
**Prior IBD related Surgery (non-perianal)**	
Diverting ileostomy	7 (37)
Subtotal colectomy	2 (10)
**Autoinflammatory manifestations**	
Recurrent fevers	19 (100)
Oral ulcers	9 (47)
Arthritis	6 (32)
Leukocytosis	19 (100)
Elevated inflammatory markers (ESR, CRP)	19 (100)
**Biochemical Markers**	
Anemia	17 (90)
Hypoalbuminemia	11 (58)
**Corticosteroid dependent**	5 (26)
**Hospitalizations 6-months prior to initiation**	16 (84)
**Malnutrition**	
Mild Malnutrition (weight/length-age or BMI Z score -1.99-(-1.0))	4 (21)
Moderate Malnutrition (weight/length-age or BMI Z score -2.99-(-2.0))	3 (16)
**Prior failed therapies, n=12**	
Infliximab	9 (75)
Vedolizumab	4 (33)
Adalimumab	4 (33)
Ustekinumab	2 (17)
Rapamycin	6 (50)
Methotrexate	5 (42)
6- Mercaptopurine	2 (17)

### Therapeutic approach

**Figure 2:**
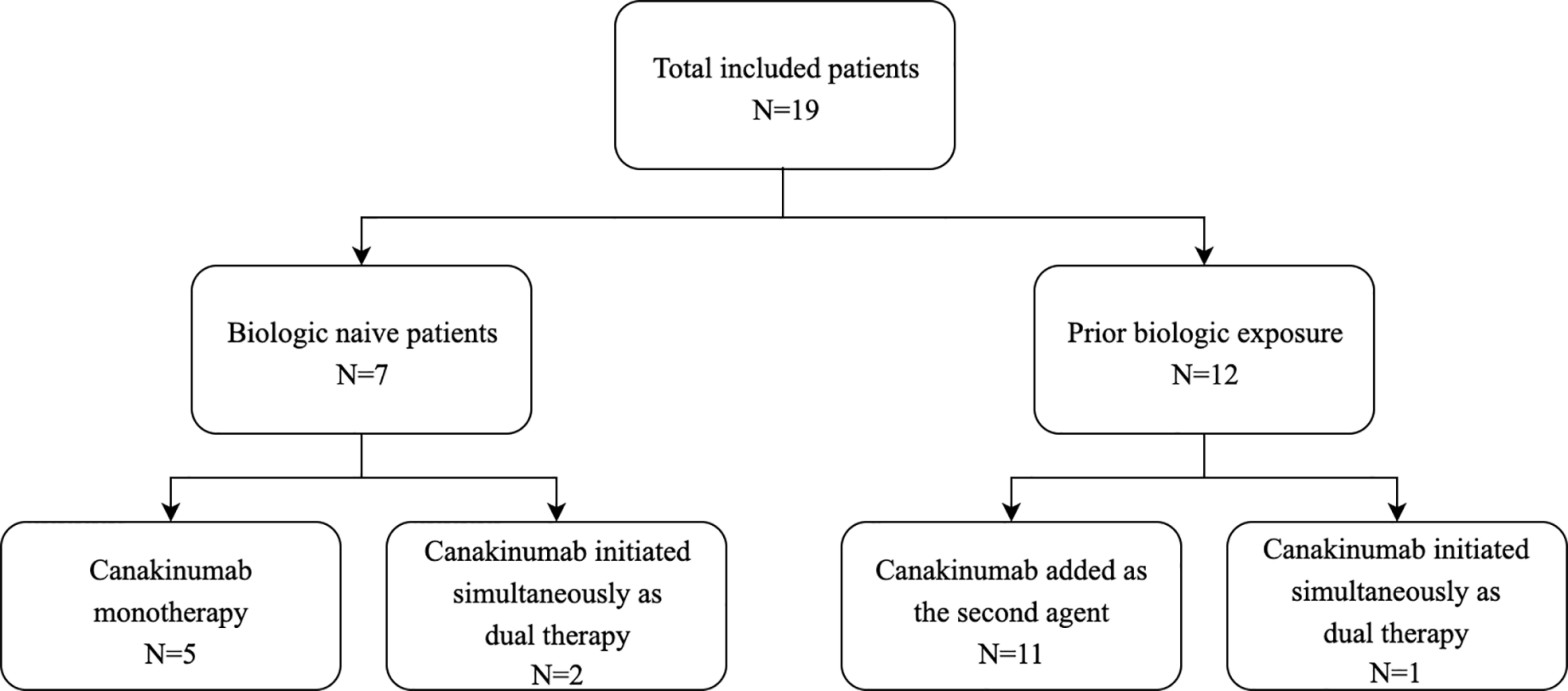
Flowchart of therapeutic approach.

Canakinumab was used in this cohort as both monotherapy and combination therapy as summarized in **Figure 2**. Canakinumab was used as the initial therapy in seven biologic-naïve patients, initiated at a median age of 0.8 years (0.3-7.25). Dual therapies used included anti-TNFα (N=4), vedolizumab (N=3), rapamycin (N=3), ruxolitinib (N=3) and ustekinumab (N=2).

### Primary outcomes

Clinical response in patients treated with canakinumab was achieved in 17/19 patients (89%) at six months. The 11 patients with IBD-U showed a statistically significant decrease in median PUCAI from 45 (25–65) at baseline to 15 (5-45; p<0.01) at six months. The eight patients with CD showed an improvement in median PCDAI from 37.5 (27.5-55) to 12.5 (5-20; p<0.05) at six months. The two patients who did not respond by six months were on dual therapy, one with vedolizumab and the other with infliximab.

At 6 months, clinical remission was achieved in six (32%) patients, all of whom had moderate-to-severe disease at baseline and four were diagnosed with infantile-onset IBD (median age of onset 3.0 months). Canakinumab was initiated within three months of diagnosis in these 4 patients. Furthermore, steroids were successfully tapered off by six months in four out of five patients who were on steroids at baseline.

### Secondary outcomes

#### Three-month clinical response and remission

Clinical indices were available for 18/19 (95%) patients at three months. Clinical response achieved in 11/18 (61%) patients with an improvement in median PUCAI from 45 (25–65) to 12.5 (5-60; p<0.01) and median PCDAI from 37.5 (27.5-55) to 10 (0-62.5) at three months (**Supplementary Table 3**). Clinical remission was achieved in 3/18 (17%) of evaluable patients.

#### Autoinflammatory features

**Figure 3:**
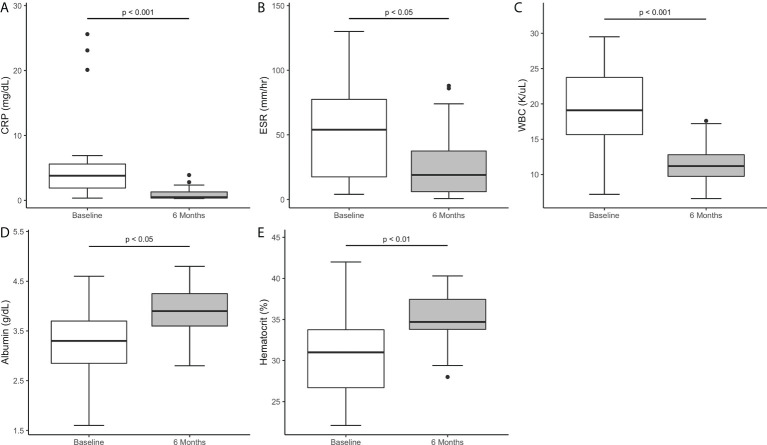
Markers of inflammation and biomarkers of disease at six months of canakinumab therapy. **(A)** CRP **(B)** ESR **(C)** WBC **(D)** Albumin **(E)** HCT.

There was a statistically significant improvement in AIP features from baseline to three and six months. This was demonstrated by a decrease in EIMs at three months, with a reduction in recurrent fevers in 15/19 patients (p<0.0001), oral ulcers in 6/9 (p<0.05) and resolution of arthritis in all six patients (p<0.05). EIMs continued to improve at six months, with 17/19 patients (p <0.0001) fever-free, resolution of oral ulcers in 7/9 patients (p<0.05), and continued resolution of arthritis (p<0.05). Moreover, there was a statistically significant decrease in the inflammatory markers as compared to baseline, both at 3 and 6 months. The median CRP decreased from 3.8 to 0.7 to 0.5 mg/dL (p< 0.01 and p<0.001), the median ESR decreased from 54 to 17.5 to 19 mm/hr (p<0.01 and p<0.05) and the median WBC decreased from 19.1 to 13 to 11.2 K/uL (p<0.01 and p<0.001) after three and six months of canakinumab therapy, respectively (**Figure 3**).

#### Biomarkers of disease activity

There was a statistically significant improvement in biomarkers of disease from baseline to three and six months with improvement in median albumin (p<0.05) and hct (p <0.01) (**Figure 3**). Furthermore, there was a statistically significant decrease in patients requiring pRBC transfusions from 11 (58%) patients in the six-month period pre-initiation to three (16%) in the six months post-initiation (p<0.01).

#### Hospitalizations

**Figure 4:**
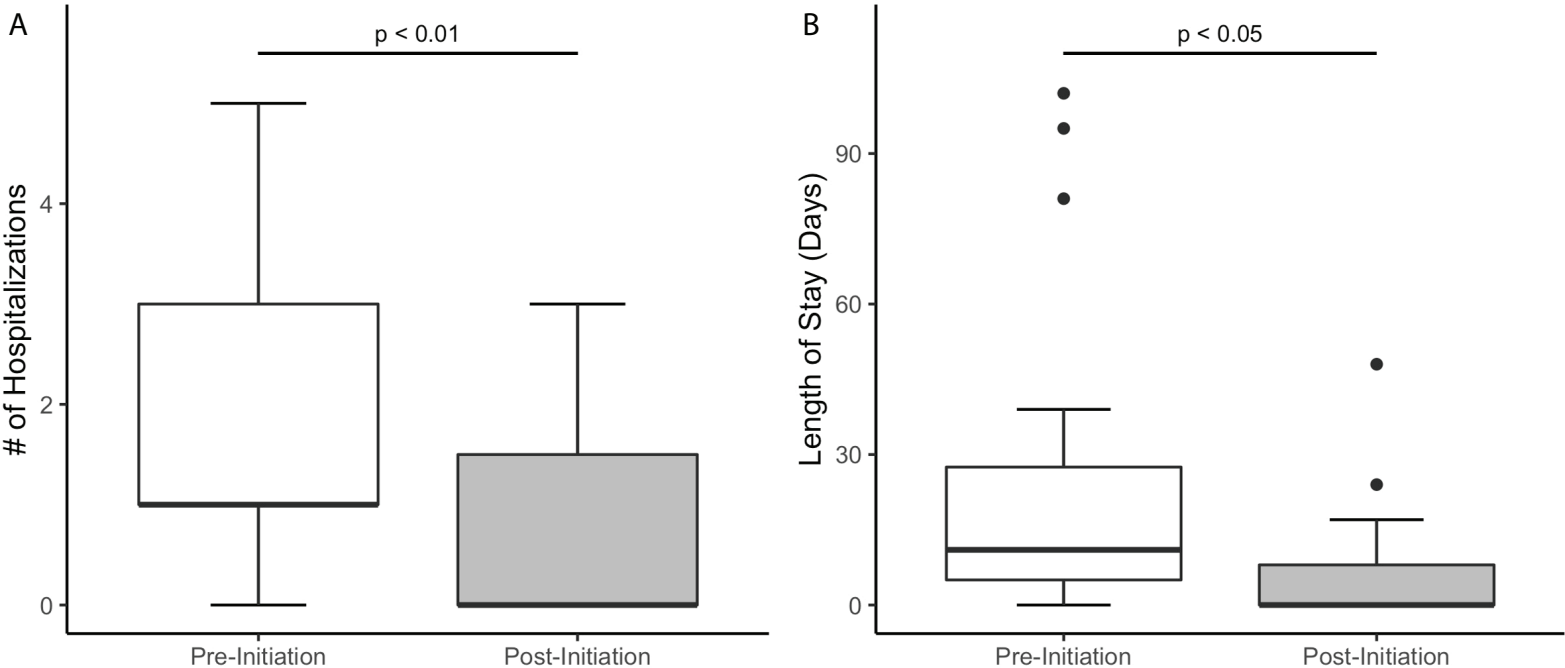
Hospitalizations six months prior and six months after initiating canakinumab therapy in patients with VEO-IBD **(A)** Decrease in number of hospitalizations **(B)** Decrease in length of stay.

Sixteen out of the 19 patients required at least one hospitalization in the six months prior to canakinumab initiation. At six-month analysis, only nine patients required hospitalization. Furthermore, the length of stay decreased from a median of 11 days (0–102) to 0 days (0-48, p<0.05) (**Figure 4**). The majority of the hospitalizations (71%) were in the setting of infectious gastroenteritis with associated dehydration. Three patients were hospitalized due to active IBD.

#### Growth

At baseline, six patients met criteria for mild malnutrition and four met criteria for moderate malnutrition. At six months there was an overall improvement in nutritional status, with resolution of malnutrition in all patients and the median weight for length Z score increased from -1.01 (-2.34-0.9) to 1.1 (0.05-1.99, p<0.01) and BMI Z score increases from 0.08 (-2.37-0.92) to 0.68 (-0.89-3.27), although it did not reach statistical significance (**Table 3**). Additionally, at baseline five patients required TPN, which was successfully discontinued in all patients by six months. Thirteen patients required supplemental NG or G tube feeds at baseline and at six months only three patients required enteral feeds (p<0.01). The indication for NG feeds for these three subjects was both nutrition and as exclusive enteral nutrition therapy.

**Table 3:** Improvement in growth parameters from baseline to six month follow up.

Growth Parameter	Median (Range)		P value
	Baseline	6 Months	
Weight-for-length for age Z Score	-1.01 (-2.34-0.91)	1.1 (0.05-1.99)	<0.01
BMI for age Z Score	0.08 (-2.37-0.92)	0.68 (-0.89-3.27)	ns
Weight for age Z score	-1.02 (-3.64-1.04)	-0.41 (-2.44-1.44)	<0.01
Length for age Z score	-1.53 (-3.93-0.4)	-1.24 (3.6-0.46)	<0.05

#### Surgical outcomes

Two of the five (40%) patients with diverting ileostomies at baseline underwent re-anastomosis after six months of therapy with canakinumab. There were no other surgeries during the study period.

#### Analysis of patients treated with canakinumab <6 months

A subset analysis was performed on the five patients with AIP VEO-IBD who were treated with canakinumab <6 months at the time of this publication and therefore did not meet study inclusion criteria. At baseline all of these patients had recurrent fevers, 3/5 had arthritis and oral ulcers present. Malnutrition was present in 4/5 patients at baseline (2 moderate, 1 severe and 1 mild). Two patients have remained on therapy at the current time. Two patients discontinued therapy due to insurance denial, and 1 patient discontinued due to parental preference.

Follow up data was obtained at 3 months for four patients, while one patient had the latest available evaluable data at 2 months. The five patients all demonstrated response as measured by PCDAI and PUCAI, as well as showed improvement in extraintestinal manifestations and laboratory studies. In addition, all four patients with malnutrition at baseline had improvement in their nutritional status. (**Supplementary Table 4**).

#### Adverse effects

Canakinumab was overall well-tolerated in this cohort. Two patients reported pain at the injection site. There were six serious events in the study cohort resulting in hospitalizations that occurred among five patients. Four hospitalizations were in the setting of infection: two patients with *Clostridioides difficile* infection and two with other causes of infectious gastroenteritis. One patient was admitted for a small bowel obstruction and underwent laparoscopic lysis of adhesions. One patient with a history of hematuria was found to have obstructive nephrolithiasis, which required laser lithotripsy and ureteral stent placement. None of the adverse events prompted discontinuation of therapy and none were clearly linked to canakinumab use.

#### Dosing

Canakinumab dosing was weight based and at the discretion of the treating clinician. The most frequent treatment dose was 5mg/kg every four weeks (range from 4mg/kg to 8mg/kg). Although the prescribed dosing frequency was four-week intervals, insurance denials led to use of eight-week intervals in seven patients. Dosing was intensified in five of these children (71%) by the third month of treatment to four-week intervals due to evidence of partial response.

## Discussion

Over the last several years, genetic discoveries in children with VEO-IBD have been transformative. However, most children remain without an identified causative defect and clear therapeutic target. Therefore, novel treatment strategies are critically needed for the significant subset of children with VEO-IBD who have severe disease with poor response to conventional IBD therapies. Phenotypic profiling to develop a targeted therapeutic approach can be an effective strategy in these children. Here, we applied this approach with canakinumab, based on its success in patients with VEO-IBD and monogenic AID (10, 20), to children with autoinflammatory VEO-IBD without identified causative defects. This study demonstrates that canakinumab is well tolerated and can be a good therapeutic option in this subset of patients. Clinical response achieved in 17/19 (89%) patients by six months; five of whom were treated with canakinumab monotherapy and 12 with dual therapy. In addition to clinical response based on standard pediatric IBD indices, we saw a statistically significant improvement in autoinflammatory characteristics, including biochemical measures of inflammation and extra-intestinal symptoms. Furthermore, canakinumab therapy led to improvement in the overall quality of life of these children with reduced hospitalizations, improvement in growth parameters, decrease in blood transfusion requirement and decreased corticosteroid use.

The use of IL-1β inhibition in AID stems from the inflammasome-mediated overproduction of caspase-1, which leads to activation of inflammatory cytokines including IL-1β and systemic inflammation. Clinically, this can manifest with symptoms of fevers, arthritis, oral ulcers, leukocytosis and elevated inflammatory markers (24). Therefore, as demonstrated in multiple studies (12, 13, 16, 25) targeted IL-1β blockade has been an effective approach in a broad spectrum of AIDs. This rationale can be expanded to its use in a subset of patients with IBD, as IL-1β plays a pivotal role in intestinal pathology by promoting neutrophil migration, T cell proliferation and IFN-γ production (26). Additionally, neutrophilic production of IL-1β results in an inflammatory response independent of caspase-1 (27). Multiple studies have shown elevated IL-1β in the GI tract in patients with IBD and showed correlation of disease activity with increased expression of IL-1β production of mononuclear cells from actively inflamed colonic mucosa as compared to non-active mucosa (8, 28). Supportive of this finding, macrophages of Nod2 knockout murine models with colitis showed increased production of IL-1β (29). Furthermore, intestinal injury in rats with acetic acid-induced colitis improved after treatment with recombinant IL-1Rα (30). Interestingly, potential mechanistic insight into the intestinal epithelial injury from IL-1β has been recently demonstrated through single cell RNA sequencing of intestinal tissue, from both humans and mice with colitis. These studies identified that elevated expression of IL- β mRNA led to increased tight junction permeability with associated inflammation (31, 32). Despite these and other animal model studies that showed improvement of colitis with IL-1α and β blockade, to date there have not been human clinical trials for IL-1 antagonists for patients with IBD. We believe this is the first study in the literature to demonstrate successful use of canakinumab therapy in children with IBD without identified causative defects.

We found that canakinumab was particularly effective when initiated as a primary therapy, early in the disease course in patients with AIP infantile-onset IBD, where robust clinical and biochemical responses were observed. Four of these patients, diagnosed under nine months of age (median 3.0 months), achieved clinical remission within three months of therapy. While larger studies are needed, this may point to positioning the use of canakinumab early in the disease course in this subset of infantile-onset IBD, potentially prior to the development of complications and sparing steroid exposure.

Similar to most therapies used for IBD, canakinumab led to clinical response when used as monotherapy and dual therapy. However, as a retrospective study with lack of controlled dosing and control arm, we recognize that the use of dual therapy in the majority of patients in this cohort (74%) may impact the interpretation of the results. Despite this limitation, it should be noted that there was significant improvement in those patients in whom canakinumab was added as the second agent without a change in dosing of the initial therapy. Due to the immense heterogeneity within the VEO-IBD population, including amongst those with AIP, the decision to use monotherapy or dual therapy was prompted by the individual patient’s disease course, including duration of disease at time of initiation of canakinumab. In both the monotherapy and dual therapy groups, consistent with the prior literature, canakinumab had an excellent safety profile with no evidence of immunogenicity and only occasional, mild injection-site pain. Although serious adverse events occurred during the study window, it is unclear whether these were directly related to canakinumab use and none of the adverse events prompted discontinuation.

The limitations of this study include the retrospective study design, lack of a comparative control group, use of dual therapy as noted above, and the small sample size. Due to the retrospective design, the dosing and use of combination therapy were at the discretion of the gastroenterologist and were not standardized. Additionally, radiologic data, cytokine studies, histopathology and fecal calprotectin were not available for all subjects pre- and post-therapy analysis, although detailed clinical activity indices and multiple biochemical markers provided for an in-depth patient evaluation.

### Conclusion

This study supports the use of canakinumab for children with autoinflammatory VEO-IBD, a population that frequently suffers from refractory severe disease. Canakinumab may be serve as an attractive alternative to other therapeutic approaches due to the narrow spectrum of immune suppression and, in our cohort, few side effects. Larger, prospective studies are needed to provide additional data regarding efficacy and safety of canakinumab in the wider VEO-IBD population. Importantly, a precision medicine approach is not limited to children with VEO-IBD. This therapy may be appropriate for treatment of older pediatric and adult IBD populations with refractory autoinflammatory phenotypes as well. The ultimate goal is to identify the driver of disease in all patients with IBD and to target these specific pathways therapeutically for optimal clinical outcomes.

## Data availability statement

The original contributions presented in the study are included in the article/**Supplementary Material**. Further inquiries can be directed to the corresponding author.

## Ethics statement

The studies involving human participants were reviewed and approved by CHOP IRB: 14-010826_AM66. Written informed consent to participate in this study was provided by the participants’ legal guardian/next of kin.

## Author contributions

JK and MC: concept. JK, ES, MC, and ND: study design. ES, SW, DA, and AB: data collection. ES, JK and ND: statistical analysis. ES and JK: drafting of the manuscript. TP, ND, MCC and AB: critical revision of the manuscript. KS, MC, JK: critical revision of the manuscript for important intellectual content. JK and MC: obtained funding. JK: study supervision. All authors contributed to the article and approved the submitted version.

## Funding

This study received funding from the NIH (NIDDK K23 DK119585, R01 DK111843 and R01DK127044-02).

## Acknowledgments

We would like to thank the entire VEO-IBD Program and collaborators, including the Children’s Hospital of Philadelphia Center for Pediatric IBD, Division of Genomic Diagnostics, and the Department of Biomedical and Health Informatics. We would also like to thank the Generous IBD Families as well as CURE for IBD for their support of the CHOP IBD Center.

## Conflict of interest

The authors declare that the research was conducted in the absence of any commercial or financial relationships that could be construed as a potential conflict of interest.

## Publisher’s note

All claims expressed in this article are solely those of the authors and do not necessarily represent those of their affiliated organizations, or those of the publisher, the editors and the reviewers. Any product that may be evaluated in this article, or claim that may be made by its manufacturer, is not guaranteed or endorsed by the publisher.

## Abbreviations

AID, Autoinflammatory diseases; AIP, Autoinflammatory phenotype; CHOP, Children’s Hospital of Philadelphia; CGD, Chronic granulomatous disease; CD, Crohn disease; CAPS, Cryopyrin-associated periodic syndromes; CRP, C-reactive protein; DHR, Dihydrorhodamine oxidation to rhodamine; EIMs, Extra-intestinal manifestations; FMF, Familial Mediterranean fever; G, Gastrostomy; hct, Hematocrit; IBD, Inflammatory bowel disease; IBD-U, Inflammatory bowel disease unclassified; IL, Interleukin; LOD, Limit of detection; MVK, Mevalonate kinase deficiency; NG, Nasogastric; pRBC, Packed red blood cell; PCDAI, Pediatric Crohn Disease Activity Index; PUCAI, Pediatric Ulcerative Colitis Activity Index; ESR, Sedimentation rate; TPN, Total parenteral nutrition; TNF, Tumor necrosis factor; UC, Ulcerative colitis; VEO, Very early onset; WBC, White blood cell.
