# Supplementary material for: Canakinumab for the treatment of autoinflammatory very early onset- inflammatory bowel disease
Source: Front Immunol. 2022 Sep 20;13:972114. doi: 10.3389/fimmu.2022.972114 (PMC9531243; doi:10.3389/fimmu.2022.972114)
Supplement: Supplementary file 4 [file Table_4.docx]

**Supplementary Table 4: Analysis of patients treated with canakinumab <6 months**

|  | **N Available at Follow-Up** | **N Improved**  **(%)** |
| --- | --- | --- |
| PUCAI | 3 | 3 (100%) |
| PCDAI | 2 | 2 (100%) |
| Fever | 5 | 4 (80%) |
| Arthritis | 3† | 3 (100%) |
| Oral Ulcers | 3† | 3 (100%) |
| CRP | 5 | 4 (80%) |
| ESR | 5 | 5 (100%) |
| WBC | 4 | 4 (100%) |
| Albumin | 4 | 3 (75%) |
| HCT | 4 | 4 (100%) |
| Malnutrition | 4‡ | 4 (100%) |

† Data was available for all 5 patients, however, only 3/5 patients had arthritis/oral ulcers at baseline

‡ Data was available for all 5 patients, however, only 4/5 patients had malnutrition at baseline
